# Exploring a Novel Technique to Tackle the Shortage of Devices for Hepatic Arterial Infusion Chemotherapy: Early Results of an Alternate Approach for Percutaneous Arterial Port Catheter Placement

**DOI:** 10.3390/cancers15194730

**Published:** 2023-09-26

**Authors:** Alice Kedra, Tom Boeken, Alessandro Di Gaeta, Charles Querub, Marc Al Ahmar, Carole Déan, Marc Sapoval, Olivier Pellerin

**Affiliations:** 1 Vascular and Oncological Interventional Radiology Department, Assistance Publique—Hôpitaux de Paris Hôpital Européen Georges Pompidou, 75015 Paris, France; tom.boeken@aphp.fr (T.B.); alessandro.digaeta@aphp.fr (A.D.G.); charles.querub@aphp.fr (C.Q.); marc.alahmar@aphp.fr (M.A.A.); carole.dean@aphp.fr (C.D.); marc.sapoval2@aphp.fr (M.S.); olivier.pellerin@aphp.fr (O.P.); 2 Faculté de Santé, Université Paris Cité, 75006 Paris, France; 3 HeKA Team, INRIA, 75015 Paris, France

**Keywords:** hepatic arterial infusion chemotherapy, percutaneous arterial port catheter, colorectal liver metastases

## Abstract

**Simple Summary:**

Dedicated catheters for hepatic arterial infusion chemotherapy were removed from the market, leading to a decrease in the number of patients eligible for this therapy. The purpose of this study was to assess the results of a novel approach to overcome the shortage of dedicated catheters for hepatic arterial infusion chemotherapy in the treatment of colorectal cancer liver metastases. The main modification of our placement technique consisted of the use of an indwelling 5-Fr Vertebral catheter, on the tip of which we created a two-sided additional lateral hole. The catheter was connected to a pediatric port. Fourteen patients underwent 15 modified procedures. The primary success rate was 100%, and the secondary success rate was 93.3%. There were two late major complications, graded IIIa according to the Clavien–Dindo classification. Our experience suggests that the derived utilization of the devices used routinely in interventional radiology provides an effective solution that can compensate for the shortage of dedicated devices.

**Abstract:**

Dedicated catheters for hepatic arterial infusion chemotherapy were removed from the market. The purpose of this study was to assess the results of a novel approach to overcome the shortage of dedicated catheters for hepatic arterial infusion chemotherapy in the treatment of colorectal cancer liver metastases. We retrospectively included patients who underwent a percutaneous placement of a hepatic intra-arterial port catheter in a single tertiary center from February 2021 to June 2022. We examined the patient baseline characteristics, technical features of the modified procedures, technical success rates, complications and oncological outcomes. Fourteen patients (median age: 60 years; q1 = 54; q3 = 70; range: 53–81 years) underwent 15 modified procedures. The main modification of our placement technique consisted of the use of an indwelling 5-Fr Vertebral catheter, on the tip of which we created a two-sided additional lateral hole. The catheter was connected to a pediatric port. The primary success rate was 100%, and the secondary success rate was 93.3%. There were two late major complications, graded IIIa according to the Clavien–Dindo classification. The median liver progression free survival was 6.1 months (q1 = 2.5; q3 = 7.2; range: 1.3–11.6). Our experience suggests that the derived utilization of the devices used routinely in interventional radiology provides an effective solution that can compensate for the shortage of dedicated devices.

## 1. Introduction

Colorectal cancer liver metastases (CRLM) are diagnosed in more than 50% of patients with colorectal cancer over the course of the disease [1]. The management of CRLM is complex and requires a multidisciplinary approach [2]. Patients with unresectable liver metastases are either treated with systemic therapies or with a combination of systemic and locoregional therapies offered by the field of interventional oncology [3].

The rationale for locoregional therapies is based on improving local disease control for patients with liver-exclusive or liver-dominant tumor burden. These include transarterial chemoembolization, selective internal radiation therapy and hepatic arterial infusion chemotherapy (HAIC). Unlike the normal hepatic parenchyma, which is primarily supplied by the portal system, liver tumors, including CRLM, are supplied by branches of the hepatic artery [4]. HAIC involves delivering chemotherapy directly into the hepatic artery, providing elevated local drug exposure with up to a 400-fold increase compared to systemic perfusion and minimizing systemic toxicities [5].

CRLM patients are referred to HAIC in various scenarios in conjunction with systemic chemotherapy. HAIC serves as an adjuvant therapy after CRLM resection or thermal ablation to address the high recurrence rate of 50% in 5 years [6,7,8], it helps convert unresectable CRLM into a resectable disease [9] and it serves as a salvage therapy for patients with unresectable CRLM. Both surgical and radiological studies have documented the technical features and specific complications of HAIC [10,11,12,13]. At the time of writing this article, over 50 registered clinical trials focusing on HAIC are recruiting patients with liver metastases or primary tumors.

Similar to venous chemotherapy, a hepatic intra-arterial catheter can be implanted and sustained, allowing for the sequential and regular administration of chemotherapy through the catheter. This method eliminates the need for repeated vascular access and hepatic artery catheterization during each session. The implantation technique was first described in the 1980s [14] and has evolved since. Hepatic intra-arterial chemotherapy is delivered through catheters placed in the hepatic artery and connected to a subcutaneously placed port. While the initial method of implantation was surgical, interventional radiologists proposed a percutaneous alternative over two decades ago, demonstrating its feasibility and safety [15].

Although evidence supporting the benefit of HAIC has grown [6], the leading manufacturer interrupted the production of a dedicated device because it could not meet the requirements of new medical regulation. Additionally, the unavailability of Celsite^®^ Anthron^®^ arterial, a dedicated catheter designed for the percutaneous delivery of locoregional chemotherapy and previously produced by B.Braun (Melsungen, Germany), has caused significant concern in the interventional radiology field and has hampered the widespread adoption of this approach. In this context, removing dedicated devices from the market could lead to a decrease in the number of patients eligible for this therapy.

To overcome the shortage of dedicated catheters for HAIC in the treatment of CRLM, the interventional radiology community has developed in-house alternatives by using available routine devices. This has helped bridge the gap until dedicated devices are more widely distributed and has enabled the continuation of HAIC for patients referred from tumor boards.

The purpose of this study was to investigate a novel solution to the shortage of specialized catheters for HAIC.

## 2. Material and Method

### 2.1. Study Population

This is a retrospective single-center IRB-approved study (IRB 00011928). The data collected were from a series of patients who underwent the procedure at a single tertiary center from February 2021 to June 2022 and were retrospectively analyzed. All the participants provided their informed consent to undergo the procedure with an implantable medical device that was used outside of its CE mark indication.

Each procedure was performed following the multidisciplinary tumor board’s decision. The patients received a consultation by the attending interventional radiologist prior to the port placement. All the interventions were performed and supervised by an interventional radiologist (OP) with experience in interventional oncology exceeding twenty years.

Patients were referred to HAIC if they met the following inclusion criteria: nonresectable liver-dominant colorectal cancer, performance status (PS) 0–1 and a vascular anatomy compatible with a port catheter placement as determined with pretreatment computed tomography (CT) with an arterial phase. No cases of adjuvant HAIC were included in the present study. Patients were not referred to HAIC if they presented liver failure, portal hypertension, a life expectancy of less than 3 months or were not available for follow-up in our center. The imaging work-up was determined by the multidisciplinary board.

The perioperative clinical and imaging data were retrospectively reviewed by an interventional radiologist with 6 years of experience. The following initial information was extracted: colorectal cancer tumor node metastasis (TNM) stage at the time of diagnosis, localization and histological type of primitive tumor, microsatellite RAS and BRAF status and number and localization of metastatic sites. The hepatic tumor burden, defined as the ratio between the tumor volume and the whole hepatic parenchyma volume at the time of intrahepatic catheter placement, was assessed visually and categorized into four classes: 0 to 25%, 25 to 50%, 50 to 75% and 75–100% of the whole liver parenchyma volume.

The technical features of the procedures and full chemotherapy protocols were reviewed. Standard clinical and imaging data obtained throughout patient care were recorded in our unified database.

### 2.2. Modified Placement Technique

**Figure 1:**
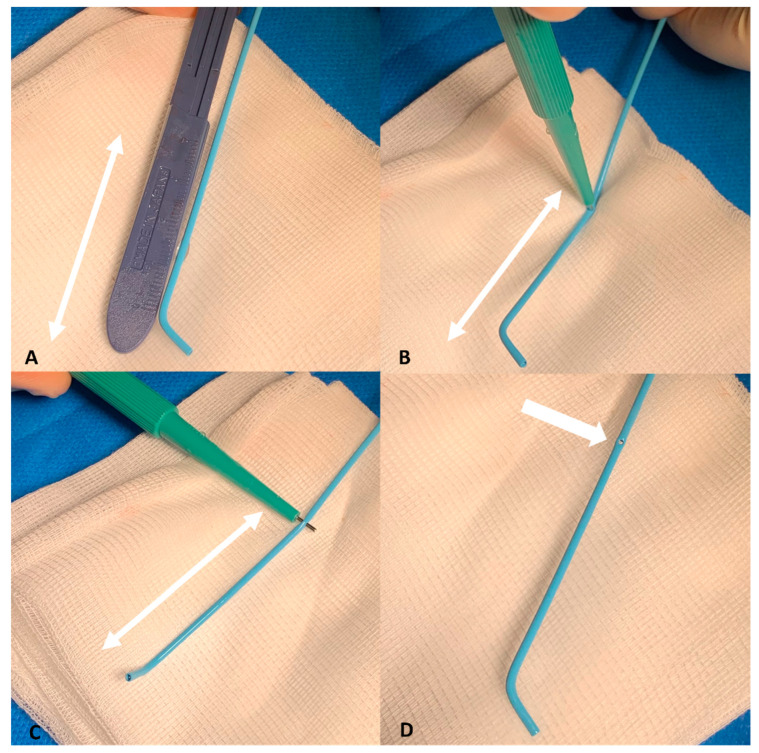
We measured the distance ((**A**), white double arrow) between the tip of the catheter and the additional two-sided 1 mm lateral hole. That distance was adjusted to the final portion of the common hepatic artery (CHA) just before the gastroduodenal artery (GDA). The additional lateral hole was made by using a 1 mm dermatological punch biopsy kit (**B**–**D**).

The replaced indwelling catheter is a mimic of the Anthron dedicated catheter. It is based on a 5-Fr, 125 cm, 0.043” inner lumen polyamide diagnostic catheter (Radifocus Angiographic Terumo Leuven B, Terumo, Shibuya, Tokyo, Japan). Before placement, this indwelling catheter is customized. A two-sided 1 mm lateral hole is created by using a dermatological punch biopsy kit. The lateral hole position is determined according to the target injection point, usually at a distance of 10–15 cm from the tip of the catheter (Figure 1).

After this initial preparation, the standard intervention is performed. All the procedures are performed in an angio suite with aseptic conditions and by using local anesthesia and prophylactic antibiotherapy (2 g of cefazolin) [16]. Initially, a 4-French (Fr) introducer sheath is placed at the femoral site by using the Seldinger technique without common femoral artery transfixion and under ultrasound guidance. Diagnostic mesenteric and celiac angiograms are performed to confirm the hepatic arterial anatomy and to assess the optimal placement of the catheter.

Before inserting the catheter, two critical objectives have to be addressed:

1. The search for the optimal injection point.
2. The skeletonization of the hepatic artery.

**Figure 2:**
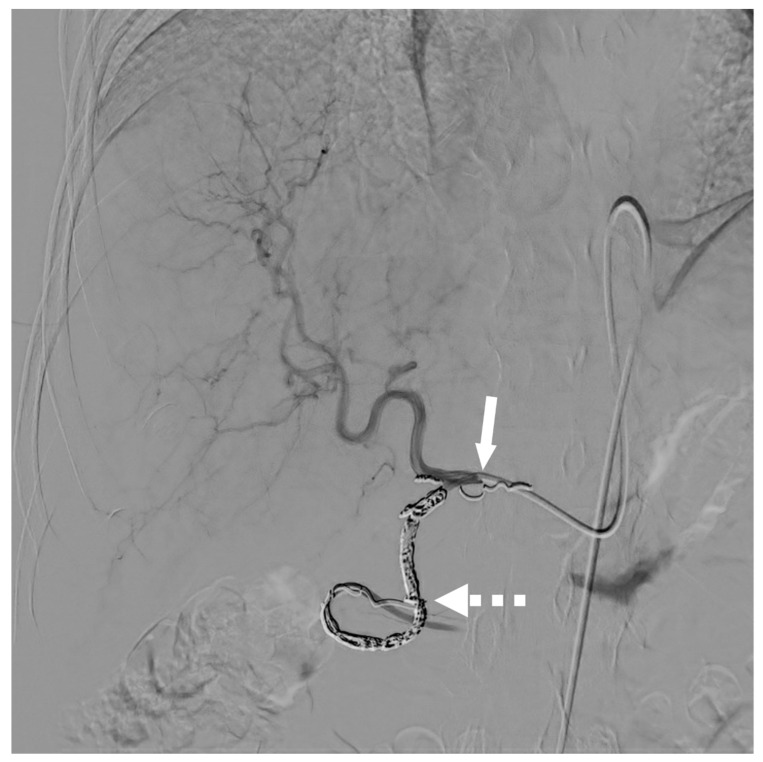
Patient treated with hepatic arterial infusion for rectosigmoid hinge cancer liver metastases. The tip of the catheter (dotted white arrow) was placed in the GDA, according to the so-called “technique 2”. The GDA was occluded thanks to coils, which also allowed us to secure the catheter. Chemotherapy was infused through the added lateral two-sided hole (white arrow).

**Figure 3:**
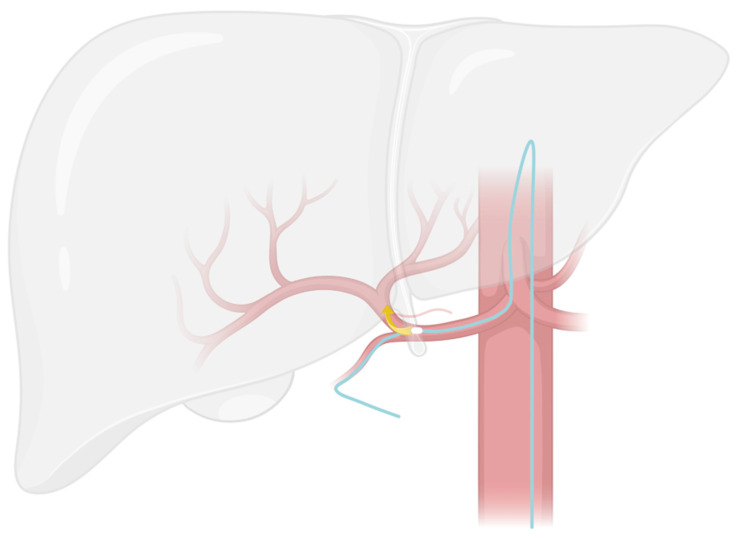
Representation of the optimal lateral hole placement and the hypothesized chemotherapy diffusion. Note the security loop within the aorta used for protection against catheter migration during daily life activities. The coils within the right gastric artery and the gastroduodenal artery are not represented for clarity.

The injection point is the ideal location in the hepatic arterial network that allows for the treatment of all the liver segments in a single session of chemotherapy without having to move the catheter. The goal is to position the side hole of the catheter at the injection point (Figure 2 and Figure 3).

Deschamps et al. [11] and Arai et al. [17] both described techniques to secure the catheter tip in the arteries. The so-called “technique 1”, consisting of putting the tip of the indwelling catheter in the common hepatic artery (CHA) after the embolization of the gastroduodenal artery (GDA) with coils, is not recommended anymore because of high migration rates. When feasible, we prefer the so-called “technique 2”: placing the tip of the catheter in the GDA and coiling it around to occlude the artery and secure the catheter. “Technique 2” is sometimes impossible to perform; for example, because the GDA was ligated, or because it did not arise from the hepatic artery where the catheter was inserted. In these cases, “technique 3” is used, consisting of putting the tip in a peripheral branch of the hepatic artery.

**Figure 4:**
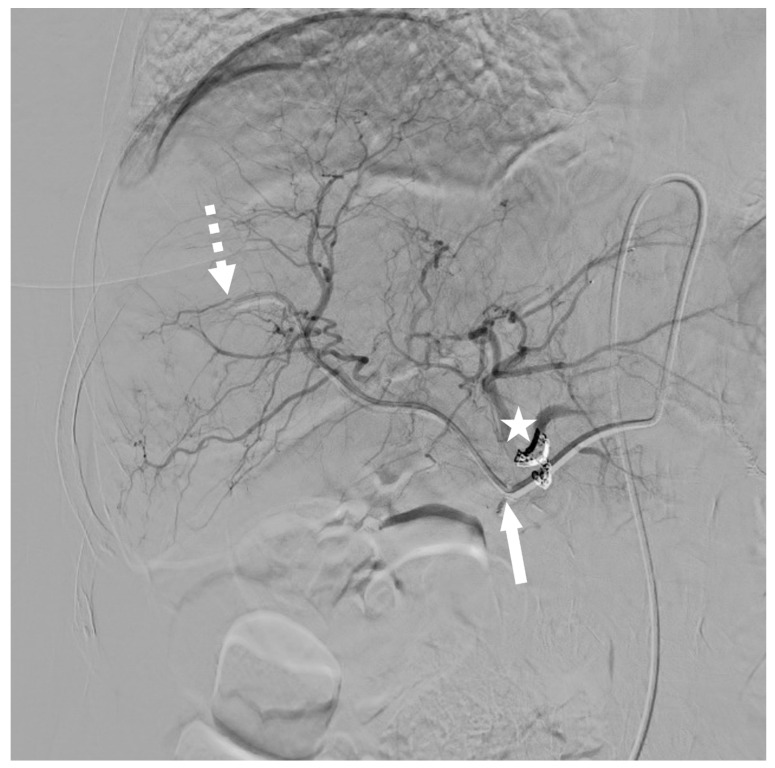
Patient treated with hepatic arterial infusion for sigmoid cancer liver metastases. The patient had a left hepatic artery (LHA) from which the GDA departed. LHA and GDA were occluded thanks to coils (white star). Vascularization of the left part of the liver was taken over by the CHA, arising from the celiac trunk, after the redistribution technique. The tip of the catheter (dotted white arrow) was placed in a peripheral branch of the proper hepatic artery (PHA) according to the so-called “technique 3”. Chemotherapy was infused through the added lateral two-sided hole (white arrow).

More specifically, the nonmodal distribution of the hepatic arterial network needs extra attention. It represents 10 to 30% of cases [18]. As it is not technically possible to implant a catheter in each arterial branch, interventional radiologists have to redistribute the hepatic arterial blood flow to ensure the treatment of all hepatic segments (Figure 4). A detailed description of intrahepatic perfusion redistribution was recently published by Kobe et al. [19]. Usually, the smallest artery is embolized while the largest one will receive the indwelling catheter. When the final position is within the replaced right hepatic artery (RHA), the catheter is placed far enough into the artery to ensure sufficient stability and, at the same time, correct perfusion to all the liver segments.

The skeletonization of the hepatic artery is the second challenge. It ensures the absence of extrahepatic chemotherapy spreading during injection. This is secured by the embolization with coils of all extrahepatic arteries arising from the hepatic arterial network with the exception of the cystic artery.

After a thorough embolization of all extrahepatic arteries, a V-18 micro guidewire (Boston Scientific, Marlborough, MA, USA) is placed distally in the right gastro-epiploic artery by using the 4-Fr catheter and a 2.4-Fr microcatheter for maximum support. Both catheters and the introducer sheath are removed. The modified 5-Fr catheter is inserted by using a coaxial 2.4-Fr microcatheter navigated on the V-18 micro guidewire. The distal catheter shaft is fixed by microcoils, usually Azur or Azur Cx (Terumo, Shibuya, Tokyo, Japan) and Interlock (Boston Scientific, Marlborough, MA, USA) through the end hole and through the side holes by using a 2.0-Fr microcatheter.

The indwelling catheter is then tunneled from the groin to the port implantation site. For that purpose, the arterial access site is previously enlarged to prevent catheter kinking and to avoid any subsequent catheter skin extrusion.

To overcome the connection issue between the port and the catheter, a pediatric port is used. The Polysite 2005 (Vygon, Ecouan, France) has a connecting hub of a 0.044” diameter that fits the 0.043” inner lumen of the modified catheter. Port insertion is made after a longitudinal skin incision (2 cm) medial to the anterosuperior iliac crest. The metallic tunneler connected to the indwelling catheter is placed at the skin puncture point made to reach the common femoral artery and pushed up into the subcutaneous fat at an approximate depth of 0.5 cm to join the skin incision. The catheter is carefully pushed 3–5 cm in the femoral artery so that it can protrude in the aorta to assess the sufficient stability. The external part of the port catheter is shortened from the incision site and connected to the port. As the catheter is not thick enough to ensure a secure grip by the olive ring, a Prolene 3.0 (Ethicon, Raritan, NJ, USA) wire loop is made and tightened over the catheter and the ring is fixed afterwards.

After the procedure of securing the port catheter, the patients are instructed to go on bedrest for 12 h.

Before the first administration of intra-arterial chemotherapy and every 4 weeks, the arterial port catheters are opacified in the angio suite with 10 mL of iodinated contrast material at a flow rate of 1.5 mL/sec by using digital subtraction angiography (DSA). Further elements are assessed: the placement of the Huber needle in the chamber, opacification of the chamber, connection between the chamber and the catheter, progression of the iodinated contrast material in the catheter, perfusion of the whole liver and lack of extrahepatic perfusion. Then, the port catheters are flushed with 20 mL of physiological serum. Intra-arterial chemotherapy is allowed if all of the previous elements are normal.

In the present study, the patients received chemotherapy cycles every two weeks. The HAIC chemotherapy was either oxaliplatin or 5-fluorouracil. Bevacizumab was interrupted four weeks prior to the implantation interventions.

### 2.3. Outcomes

Primary success was defined as the immediate technical success of the port catheter placement procedure, from the initial arterial puncture to the closure of the skin incision protecting the chamber. Secondary success was defined as functional success, involving the possibility of intra-arterial chemotherapy administration either directly after the procedure or after revisions.

The occurring complications were graded by using the Clavien–Dindo scale [20]. Complications were specified as early complications (<1 month), late complications (>1 month) or catheter dysfunctions. Complications related to the implantation included hematomas, arterial dissections and pseudoaneurysms, and complications associated with catheter use included infections, biliary complications and gastroduodenal ulcers due to extrahepatic chemotherapy infusion. In the case of infections persisting after antibiotics administration, the port catheter was removed in aseptic conditions under local anesthesia.

The dysfunction of the catheter included catheter thrombosis, catheter migration, extrahepatic perfusion or incomplete hepatic perfusion or disconnection between the port catheter and the indwelling catheter. Catheter thrombosis was treated by the perfusion of 2 mg of alteplase. In the case of catheter migration, an additional procedure to correct the catheter position was performed. In the case of an unmanageable catheter occlusion or migration, the port catheter was removed in aseptic conditions under local anesthesia. If the patient noticed abdominal pain during the chemotherapy infusion, the opacification of the port catheter was performed in the angio suite to check if there was any extrahepatic perfusion. If an extrahepatic branch was opacified through the port catheter, a new embolization was planned, usually via left radial access.

The oncological outcomes included a hepatic tumor response and global oncologic response according to the response evaluation criteria in solid tumors (RECIST) 1.1 on the latest CT control, hepatic progression-free survival (PFS), overall PFS and overall survival (OS).

### 2.4. Statistical Analysis

The statistical analysis was carried out by using version 4.0.3 of R software. If considered normally distributed (with a Shapiro–Wilk test), the continuous variables were described by using the mean and standard deviation and were then compared by using two-sided *t*-tests. Other variable types were characterized by using the median as well as the first and third quartile and range [21]. No complex statistical analysis was performed for this study.

## 3. Results

### 3.1. Study Population

A total of 14 consecutive patients, 8 men and 6 women, with a median age of 60 years (q1 = 54; q3 = 70; range: 53–81) were treated between February 2021 and June 2022 in our tertiary center. They underwent 15 interventions. The baseline characteristics of our study population are detailed in Table 1.

In total, 9 of the 14 patients (64.3%) had synchronous liver metastases whereas 5/14 (35.7%) had metachronous disease. The median time lapse between the initial diagnosis and intra-arterial catheter implantation was 32.2 months (q1 = 21.4; q3 = 57.2; range: 0.7–161.7). At the time of intra-arterial catheter implantation, hepatic tumor burden was estimated between 0% and 25% of the whole liver parenchyma in 10/14 cases (71.4%), between 25% and 50% in 3/14 cases (21.4%) and between 50% and 75% in 1/14 cases (7.1%). The most common extrahepatic metastatic site was the lung (6/14, 42.9%), followed by extraregional nodes (2/14, 14.3%). The hepatic arterial anatomy, determined with pretreatment CT with the administration of iodinated contrast material at the arterial phase and confirmed on the per procedural angiogram, was modal according to the Michels classification [18] in 8/14 cases (57.1%). For one of these patients, their hepatic arterial anatomy was modal because of a previous intra-arterial catheter implantation performed many years ago, during which the left hepatic artery (LHA) was embolized to allow the perfusion of the whole hepatic parenchyma by a single artery. The main anatomical variants consisted of the presence of a replaced LHA in 4/14 cases (28.6%), a replaced RHA in 2/14 cases (14.3%) and an arising GDA from the left branch of the proper hepatic artery (PHA) in 2/14 cases (14.3%).

### 3.2. Modified Implantation Technique

Intra-arterial catheter placement was performed by using the so-called “technique 2” according to the Deschamps classification [11] in 5/14 (33.3%) cases. “technique 3” was used in 10/14 cases (66.7%). This latter technique was usually employed when the anatomical configuration of the GDA did not allow for the insertion of the indwelling catheter into it. This occurred when the whole liver was perfused by the replaced RHA (no GDA rising from the main hepatic artery) in 2/14 cases, when the GDA arose from the left branch of the PHA and both of them were embolized so that the liver could be perfused only by the right branch of the PHA in 2/14 cases, when the GDA was already occluded (previous embolization or surgery) in 2/14 cases or when the GDA had a smaller caliber than the indwelling catheter in 1/14 cases.

In order to achieve hepatic artery skeletonization, we had to embolize 4/14 replaced LHAs; 1/14 CHA (in this case, the vascularization of the liver was ensured by the replaced RHA); and in 2/14 cases, the left branch of the PHA since the GDA arose from it.

The median duration of the intervention was 90 min (q1 = 74.5; q3 = 110; range: 35–195) with a median dose area product (DAP) of 13,232.8 µGy.m^2^ (q1 = 1827.2; q3 = 23,939.5; range: 743.3–21,000,000). The shortest duration corresponded to the second procedure for a patient who underwent the first procedure, during which the operator had already embolized the RGA and the GDA. This allowed us to shorten the second procedure.

### 3.3. Technical Outcomes

Fifteen procedures were performed for the 14 patients, with a primary success rate of 100%. Secondary success was obtained in 14/15 cases (93.3%). One patient experienced early dysfunction of the port catheter (a disconnection between the chamber and the catheter), associated with an infection, which led to its ablation before any cycle of HAIC. A second port catheter could be implanted after two weeks of a well-conducted antibiotic treatment.

The patients received a median of seven cycles of HAIC (q1 = 5.25; q3 = 12.75; range: 3–22). Ten (71.4%) patients received intra-arterial oxaliplatin and five patients (35.7%) received intra-arterial 5-fluorouracil: one patient had 5-fluorouracil after oxaliplatin because of delayed hypersensitivity.

There was no complication directly related to the 15 implantation procedures such as hematoma, arterial dissection or false aneurysm. There were 2/14 (14.3%) late complications that were graded IIIa according to the Clavien–Dindo classification, corresponding to 0.082 complications per 100 catheter days [20]. One patient encountered an early disconnection between the chamber and the catheter (1 week after the first procedure), which required an additional procedure to reconnect both elements. Then, the patient developed an infection due to the port catheter that was resistant to antibiotics at 1.5 months after the procedure, which led to the removal of the device and to a second implantation procedure 3 weeks later. Another patient presented with a perforated gastroduodenal ulcer 6.5 months after the procedure due to the extrahepatic infusion of chemotherapy. We encountered a total of 0.34 dysfunctions per 100 catheter days: 1/14 thrombosis (7.1%), 1/14 migration of the catheter (7.1%), 2/14 extrahepatic perfusions (14.3%), 1/14 incomplete hepatic perfusion (7.1%) and 2/14 disconnections between the chamber and the catheter (14.3%). These dysfunctions occurred mostly early after the implantation (5/7, 71.4% before one month), between 1 week and 3.5 months after the first procedure, with a median of 1 month. All the dysfunctions were treated successfully. The catheter thrombosis was treated with 2 mg of alteplase. The migrated catheters and extrahepatic perfusions were managed with an additional intervention.

### 3.4. Oncological Outcome

The median follow-up from the implantation was 7.9 months (q1 = 4.8; q3 = 10.6; range: 1.4–15.2).

On the last control CT according to RECIST 1.1, 1/14 patients (7.1%) had a complete hepatic response: the patient had an important partial response and underwent a right hepatectomy without progression on the remaining hepatic parenchyma. In total, 3 patients (21.4%) presented a partial hepatic response, 4/14 patients (28.6%) presented a stable hepatic disease and 5/14 patients (35.7%) had liver progression. The median hepatic PFS was 6.1 months (q1 = 2.5; q3 = 7.2; range: 1.3–11.6) and the median overall PFS was 4.8 months (q1 = 2.5; q3 = 6.1; range: 1.3–11.6).

The median OS from the date of the implantation was 7.7 months (q1 = 4.2; q3 = 9.8; range: 1.4–11.7). Five patients died during the follow-up period, four because of end-stage disease evolution and one patient because of hepatocellular dysfunction secondary to hepatectomy. No mortality was associated with the port catheter implantation.

## 4. Discussion

We report the initial experience of a modified technique aimed at overcoming the shortage of the dedicated catheter. This specific device is a hydrophilic polyurethane catheter to which heparin is ionically bound and is particularly used to help prevent catheter occlusion and catheter-related thrombosis. It is a tapered 5-Fr proximal and 2.7-Fr distal catheter with a side hole located 20 cm from the 2-Fr extremity. We report in this study a mimic of the dedicated device based on available solutions.

The present study shows a primary success rate of the modified port catheter implantation technique of 100% and a secondary success rate of 93.3%. The only patient whose first procedure ended with the port catheter removal before the administration of any HAIC cycle underwent another procedure, after which intra-arterial chemotherapy could be administered. These results are similar to those observed in other studies, especially studies published before the shortage of the dedicated device [22]. Additionally, the complication rates are also comparable to published data [23,24,25,26]. These elements suggest that the modified technique is feasible to assess HAIC even without the dedicated device.

The high rate of catheter dysfunction confirms the need for the continuous and intensive management of patients treated with HAIC; in each case, the catheter dysfunctions were treated with procedures that permitted us to continue the HAIC cycles.

The catheter dysfunctions are mostly due to the complex arterial anatomy and to the lack of stability of the catheter. The stability of the catheter is hard to predict and often requires further adjustments. Some authors recommended the use of n-butyl cyanoacrylate (NBCA) mixed with Lipiodol to fix the indwelling catheter into the GDA [24], but this technique remains controversial because of a potential risk of nontarget embolization and of NBCA migration in the case of port catheter removal. At a time when the device initially used is no longer available, there may be room for inventing new techniques more adapted to the anatomy of the hepatic arteries. For example, shapeable catheters could be used in order to adapt the shape of their tip to the shape of the artery branch in which they will be anchored.

Nevertheless, the observed catheter dysfunctions may be associated with the modified implantation technique. For example, we encountered two disconnections between the chamber and the catheter: at the beginning of the modified procedure learning curve in April and June 2021. This may be explained by the fact that the chamber employed is not designed to be connected to the 5-Fr Vertebral catheter. After performing several modified procedures, it was decided to reinforce the connection between both elements by fastening the catheter to the chamber by using 3.0 Prolene and clipping the ring afterwards. Later on, there was no additional disconnection between the chamber and the catheter.

Another concern was the lack of heparin coating on the customized catheter. This may partially explain the high rate of catheter thrombosis. The rate (6.7%) is yet consistent with the rates found in the literature, including surgical and percutaneous implantation techniques (6.0–11%) [11,13].

The oncological results must be interpreted with caution since the main objective of this study was to evaluate the feasibility and security of the modified implantation technique and the present work was not designed to evaluate the treatment efficiency. The population is very heterogeneous mostly because of the time lapse between the diagnosis and the port catheter implantation. Some patients were proposed HAIC after more than ten years of different treatment lines, and HAIC appeared to be a salvage therapy. On the contrary, some patients were included shortly before the end of the follow-up time, and the disease response according to RECIST 1.1 is difficult to interpret in these cases. However, it is interesting to note that HAIC allowed for local hepatic control in several cases. This is relevant since the vital prognosis of colorectal cancer patients, especially at the end stage, depends on the liver disease [27,28,29].

Our study has certain limitations that must be considered. Firstly, it is a retrospective, single-center study that was conducted on a limited patient population, and as such, its results may not be representative of a broader population. Secondly, the relatively short follow-up time may not provide a complete picture of the long-term complications associated with the modified technique. Further, large-scale, multicenter studies with longer follow-up periods are necessary to fully assess this concern and provide a more comprehensive understanding of the modified technique’s long-term outcomes.

## 5. Conclusions

In conclusion, our proposed solution involves utilizing routine catheters to address the shortage of specialized devices for HAIC. While this approach offers a viable alternative, further long-term monitoring and evaluation are needed to establish its efficacy and widespread adoption. Until more advanced and dedicated devices become readily available for patients with CRLM, our proposed method may provide a practical palliative solution.

## Figures and Tables

**Table 1:** Initial characteristics of the study population.

Variable		*N* (%) or Median (Range)
Gender	Male	8 (57.1)
	Female	6 (42.9)
Age		60 (53–81)
PS *	0	8 (57.1)
	1	6 (42.9)
Time lapse from diagnosis to hepatic intra-arterial catheter implantation (months)		32.2 (0.7–161.7)
Localization of primitive tumor	Rectum	3 (21.4)
	Rectosigmoid hinge	1 (7.1)
	Sigmoid	6 (42.9)
	Left colon	1 (7.1)
	Right colon	1 (7.1)
	Cecum	2 (14.3)
Histological type	Adenocarcinoma	14 (100)
MSS/MSI Status	MSS	14 (100)
	MSI	0 (0)
RAS Status	Wild type	6 (42.9)
	Mutated	8 (57.1)
BRAF Status	Wild type	14 (100)
	Mutated	0 (0)
Number of metastatic sites (liver included) *	1	6 (42.9)
	2	8 (57.1)
Extrahepatic metastatic site *	Extraregional node	2 (14.3)
	Lung	6 (42.9)
	Kidney	1 (7.1)
Hepatic burden *	0–25%	10 (71.4)
	25–50%	3 (21.4)
	50–75%	1 (7.1)
	75–100%	0 (0)
Previous surgical treatment of primitive tumor		10 (71.4)
Previous surgical or radiological treatment of liver metastases	Yes	9 (64.3)
	Surgical	2 (14.3)
	Interventional Radiology	3 (21.4)
	Both	4 (28.6)
Number of systemic therapy lines before HAIC	0	1 (7.1)
	1	2 (14.3)
	2	6 (42.9)
	3	0 (0)
	4	4 (28.6)
	>4	1 (7.1)
Hepatic arterial anatomy	Modal	8 (57.1)
	Variant	6 (42.9)
Type of anatomical variants	LHA	4 (28.6)
	RHA	2 (14.3)
	GDA rising from left branch of PHA	2 (14.3)

* indicates outcome at the time of hepatic intra-arterial catheter implantation. HAIC refers to hepatic arterial infusion chemotherapy. LHA refers to left hepatic artery. RHA refers to right hepatic artery. GDA refers to gastroduodenal artery. PHA refers to proper hepatic artery.

## Data Availability

The data that support the findings of this study are available from the corresponding author (A.K.) upon reasonable request.

## Abbreviations

CHA	common hepatic artery
CLRM	colorectal liver metastases
CT	computed tomography
DAP	dose area product
DSA	digital subtraction angiography
Fr	French
GDA	gastroduodenal artery
HAIC	hepatic arterial infusion chemotherapy
IRB	institutional review board
LGA	left gastric artery
LHA	left hepatic artery
NBCA	n-butyl cyanoacrylate
OS	overall survival
PFS	progression-free survival
PHA	proper hepatic artery
PS	performance status
RECIST	response evaluation criteria in solid tumors
RGA	right gastric artery
RHA	right hepatic artery
TNM	tumor node metastasis
